# Understanding the Role of SARS-CoV-2 ORF3a in Viral Pathogenesis and COVID-19

**DOI:** 10.3389/fmicb.2022.854567

**Published:** 2022-03-09

**Authors:** Jiantao Zhang, Amara Ejikemeuwa, Volodymyr Gerzanich, Mohamed Nasr, Qiyi Tang, J. Marc Simard, Richard Y. Zhao

**Affiliations:** ^1^Department of Pathology, University of Maryland School of Medicine, Baltimore, MD, United States; ^2^Research and Development Service, VA Maryland Health Care System, Baltimore, MD, United States; ^3^Department of Neurosurgery, University of Maryland School of Medicine, Baltimore, MD, United States; ^4^Drug Development and Clinical Sciences Branch, Division of AIDS, NIAID, NIH, Bethesda, MD, United States; ^5^Department of Microbiology, Howard University College of Medicine, Washington, DC, United States; ^6^Department of Microbiology and Immunology, University of Maryland School of Medicine, Baltimore, MD, United States; ^7^Institute of Human Virology, University of Maryland School of Medicine, Baltimore, MD, United States; ^8^Institute of Global Health, University of Maryland School of Medicine, Baltimore, MD, United States

**Keywords:** SARS-CoV-2, COVID-19, ORF3a, viral pathogenesis, virus–host interaction

## Abstract

The ongoing SARS-CoV-2 pandemic has shocked the world due to its persistence, COVID-19-related morbidity and mortality, and the high mutability of the virus. One of the major concerns is the emergence of new viral variants that may increase viral transmission and disease severity. In addition to mutations of spike protein, mutations of viral proteins that affect virulence, such as ORF3a, also must be considered. The purpose of this article is to review the current literature on ORF3a, to summarize the molecular actions of SARS-CoV-2 ORF3a, and its role in viral pathogenesis and COVID-19. ORF3a is a polymorphic, multifunctional viral protein that is specific to SARS-CoV/SARS-CoV-2. It was acquired from β-CoV lineage and likely originated from bats through viral evolution. SARS-CoV-2 ORF3a is a viroporin that interferes with ion channel activities in host plasma and endomembranes. It is likely a virion-associated protein that exerts its effect on the viral life cycle during viral entry through endocytosis, endomembrane-associated viral transcription and replication, and viral release through exocytosis. ORF3a induces cellular innate and pro-inflammatory immune responses that can trigger a cytokine storm, especially under hypoxic conditions, by activating NLRP3 inflammasomes, HMGB1, and HIF-1α to promote the production of pro-inflammatory cytokines and chemokines. ORF3a induces cell death through apoptosis, necrosis, and pyroptosis, which leads to tissue damage that affects the severity of COVID-19. ORF3a continues to evolve along with spike and other viral proteins to adapt in the human cellular environment. How the emerging ORF3a mutations alter the function of SARS-CoV-2 ORF3a and its role in viral pathogenesis and COVID-19 is largely unknown. This review provides an in-depth analysis of ORF3a protein’s structure, origin, evolution, and mutant variants, and how these characteristics affect its functional role in viral pathogenesis and COVID-19.

## Introduction

### SARS-CoV-2 and Genome Organization

Severe acute respiratory syndrome coronavirus 2 (SARS-CoV-2) is an enveloped, positive-sense, single-stranded RNA (+ssRNA) virus that belongs to the genus *Betacoronavirus* of *Coronaviridae* family (Coronaviridae Study Group of the International Committee on Taxonomy of Viruses, 2020). SARS-CoV-2 is one of the 7 human coronaviruses (hCoVs) found in α-CoV and β-CoV that cause human diseases ranging from the common cold (229E, NL63, OC43, and HKU1) to severe diseases including SARS (Severe acute respiratory syndrome), MERS (Middle East respiratory syndrome) and coronavirus disease 2019 (COVID-19). SARS-CoV, MERS-CoV, and SARS-CoV-2 are β-CoVs. In its five subgenera or lineages (A, B, C, D, and E), SARS-CoV and SARS-CoV-2 belong to the B lineage known as Sarbecovirus. MERS-CoV is in the C lineage, *a.k.a*. Merbecovirus (Woo et al., 2010; Boni et al., 2020).

**Figure 1:**
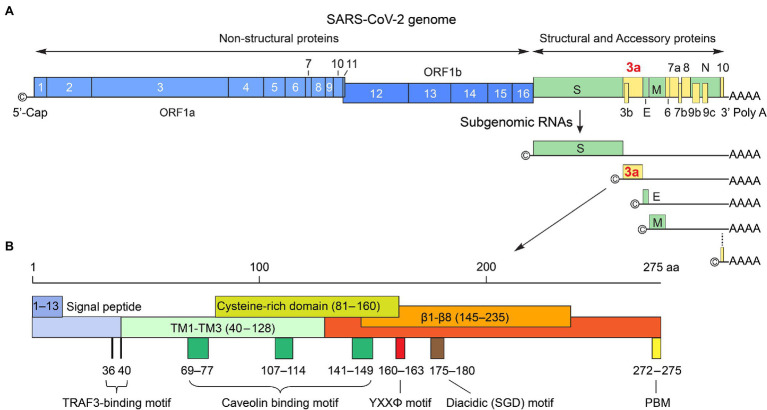
SARS-CoV-2 genome organization and ORF3a protein. **(A)** SARS-CoV-2 RNA genome organization. The genome size is about 29.7 kb of non-segmented, +ssRNA. The 5′ cap structure and 3′ poly (A) tail are for translation of ORF1a and ORF1ab that are further processed to generate 16 non-structural proteins (NSP1-NSP16) that include all major replicase genes and enzymes that are needed for subsequent viral transcription and replication. The sgRNAs produced in RTC on DMVs are for 4 structural proteins (S, E, M, and N) and 9 accessary ORFs (3a, 3b, 6, 7a, 7b, 8, 9b, 9c, and 10). These proteins are produced from a nested set of sgRNAs that share common 3′ ends and a common leader from the 5′ end. ORF3a is the largest accessory protein. It localizes between S and E as shown. **(B)** The nucleotide sequence of *ORF3a* is 825 bp in length and encodes a 31 kD protein of 275 a.a. It is a membrane-associated protein with an extracellular N-terminal (a.a. 1–39), 3 TM domains (TM1-TM3; a.a. 40–128); a short cytoplasmic loop (a.a. 175–180) with 8 β-sheets (β1–β8; a.a., 145–235) and a C-terminus (a.a. 239–275). Known and well-conserved functional motifs are depicted; details of these motifs are described in text, including an N-terminal signal peptide (a.a. 1–13); a TRAF3-binding motif (a.a. 36–40; Siu et al., 2019; Jin et al., 2021); a cysteine-rich domain (a.a. 81–160; Lu et al., 2006); 3 caveolin-binding motifs (a.a. 69–77, 107–114 and 141–149; Padhan et al., 2007); a YXXΦ motif (a.a. 160–163) and a diacidic (SGD) motif (a.a. 175–180; Tan et al., 2004; Minakshi and Padhan, 2014), and a PBM (a.a. 272–275; Castano-Rodriguez et al., 2018; Caillet-Saguy et al., 2021).

SARS-CoV-2 genome is about 29.7 kb (Figure 1A). It contains a 5′ cap structure and a 3′ poly (A) tail, allowing direct translation of replicase proteins from genomic RNA (gRNA). The viral genome encodes a total of 29 CoV-2 proteins including 16 nonstructural proteins (NSP1-NSP16), 4 structural proteins, spike (S), envelope (E), membrane (M) and nucleocapsid (N), and 9 accessary ORFs (3a, 3b, 6, 7a, 7b, 8, 9b, 9c, and 10; Gordon et al., 2020). Among them, ORF3a, ORF8, ORF9c, and ORF10 are unique to SARS-CoV-2 (Gordon et al., 2020). However, transcriptomic and proteomic analyses of the SARS-CoV-2 genome showed very low expression levels of ORF3b, ORF9b, and ORF10 (Bojkova et al., 2020; Davidson et al., 2020; Finkel et al., 2021). About two-thirds of the 5′ terminal genome, comprised of the two overlapping ORF1a and ORF1b, encode NSPs including all the major replicase genes and enzymes. The other one-third of the viral genome from the 3′ terminal genome produces four structural proteins with all the accessory proteins imbedded among them. The structural proteins and accessory proteins are expressed from a nested set of subgenomic RNAs (sgRNAs) that are made up of intermediate negative RNAs and share common 3′ ends and a common leader from the 5′ end of sgRNA (Liu et al., 2014; Sola et al., 2015). ORF3a, residing between S and E proteins, is the largest accessory protein (Rota et al., 2003; Figure 1A).

### SARS-CoV-2 Infectious Life Cycle

**Figure 2:**
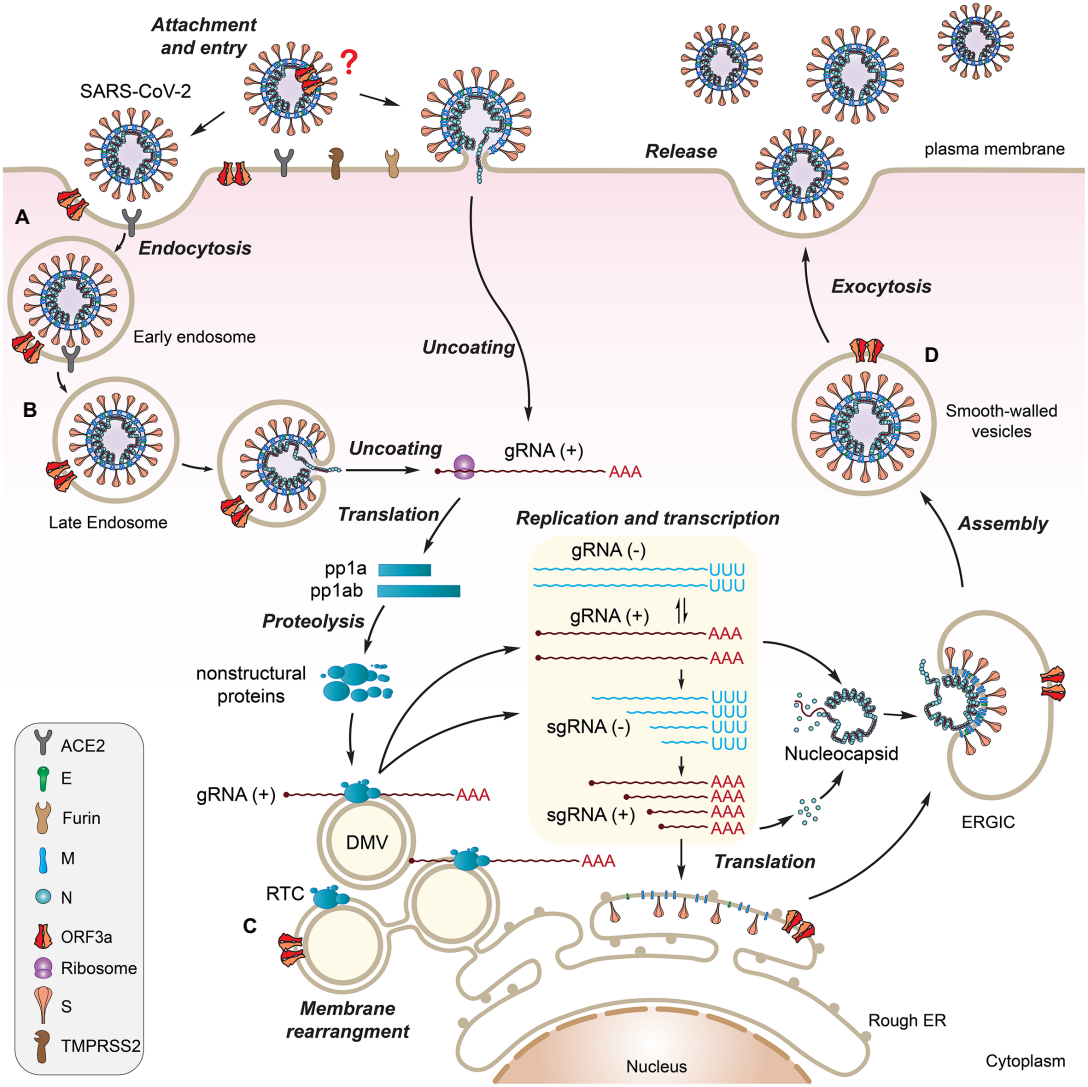
Possible involvement of SARS-CoV-2 ORF3a during viral life cycle. SARS-CoV-2 viral life cycle starts with the attachment of a virion to a host cell surface receptor ACE2 *via* S protein **(A)**. A conformational change in S induces virus membrane fusion with either the host plasma membrane or the endosome membrane, leading to the release of viral RNA into the cytoplasm **(B)**. ORF3a localizes on plasma membrane where it forms Ca^2+^ ion channel or it may interact with S protein to promote virus uptake. It also associates with early, late endosomes and lysosomes to facilitate endocytosis **(C)**. Upon viral entry, genomic +ssRNA is uncoated to allow direct translation of polyproteins, transcription of sgRNAs, and replication of the viral genome through RTC on DMVs, where ORF3a may exert its effect **(C)**. Newly produced envelope proteins are inserted into the rough ER membranes. Nucleocapsid proteins bind to genomic +ssRNA to form nucleocapsids. After budding into the ER–Golgi intermediate compartment (ERGIC), the virions are matured in smooth-walled vesicles and subsequently released from infected cell *via* exocytosis **(D)**. ORF3a promotes viral release through lysosomal exocytosis pathway. This figure is generated using Adobe Illustrator 2020.

SARS-CoV-2 infects host cells through the binding of S protein to a specific host cell surface receptor, angiotensin-converting enzyme 2 (ACE2; Hoffmann et al., 2020). Upon proteolytic cleavage by a host protease TMPRSS2, a conformational change in S protein takes place that triggers virus membrane fusion with the host cell membrane, resulting in the release of the nucleocapsid into the cytoplasm. After the virus enters the host cell, viral gRNA is uncoated and released into cytoplasm where the two overlapping *ORF1a* and *ORF1b* are translated to produce two polyproteins *via* a frameshift mechanism. One unique feature of β-CoVs reproduction is that its structural proteins and accessory proteins are produced from a replication–transcription complex (RTC), which drives viral replication and transcription for virus reproduction (Knoops et al., 2008). The RTC is formed by the action of NSP3/4/6 in SARS-CoVs (Angelini et al., 2013; Hagemeijer et al., 2014; Oudshoorn et al., 2017), which binds to the membranes of endoplasmic reticulum (ER) to induce membrane curvature, forming unique double-membrane vesicles (DMVs) where RTC resides. During viral genome replication, full-length and positive-sense genomic RNA (+gRNA) in RTC is used as a template to generate full-length and negative-sense gRNAs (-gRNA) that subsequently serve as a template to produce progeny of +gRNA. During viral transcription, a nested set of sgRNAs is produced using +gRNA as a template in a manner of discontinuous or fragmented transcription. Then, -sgRNAs are used as templates to produce +sgRNAs, which act as mRNA for translation of structural proteins and accessory proteins. Even though these sgRNAs have multiple ORFs, only the closest gene-encoding ORF (to the 5′ end) will be first translated and followed by others in a sequential order (Figure 1A). Following the production of structural proteins, nucleocapsids are assembled in the cytoplasm, followed by budding into the lumen of the ER–Golgi intermediate compartment (ERGIC). The newly generated virions are matured in smooth-walled vesicles and subsequently released (egress) from infected cell *via* exocytosis (Figure 2).

### Open Reading Frame 3a

**Figure 3:**
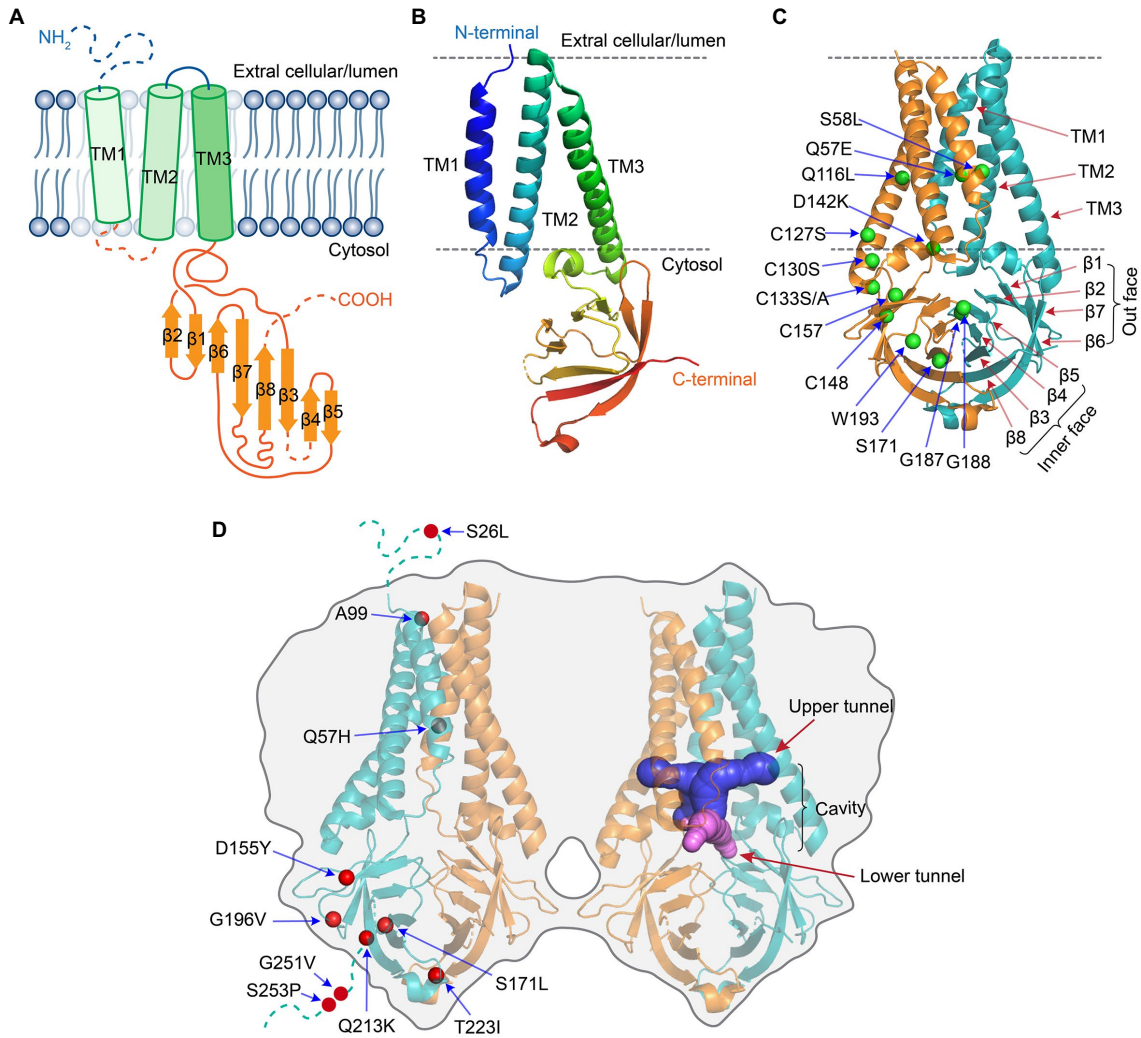
Protein structure of SARS-CoV-2 ORF3a [adapted from (Kern et al., 2021)]. **(A)** Schematic drawing of a monomeric ORF3a. It shows an extracellular N-terminus, 3 TM domains that are across the cellular membrane followed by a short cytoplasmic loop of turn-helix-turn that connects the 3-TM domains with 8 antiparallel β-sheets at the cytoplasmic C-terminus. **(B)** Monomeric ORF3a in 3D view. **(C)** Dimeric view of ORF3a. One monomer is colored teal, the other orange. Labeled a.a. residues are artificial mutations that are known affect the ORF3a protein structure or function. **(D)** Tetramer view of ORF3a showing two homodimers linked side-by-side. Labeled a.a. residues are natural mutations that are mostly of unknown impact on ORF3a protein structure. The protein 3D structure of ORF3a (PDB: 7KJR) was visualized with PyMol. All the images were generated using Adobe Illustrator 2020.

The ORF3a protein was initially uncovered from a family of coronaviruses, and it was subsequently described under different names including X1 (Rota et al., 2003), 3a protein (Marra et al., 2003; Yu et al., 2004), U274 (Tan et al., 2004), and ORF3a (Padhan et al., 2007). The potential importance of ORF3a in viral pathogenesis emerged with the discovery of SARS-CoV in 2003 followed by the emergence of SARS-CoV-2. SARS-CoV and SARS-CoV-2 ORF3a proteins (hereafter referred as SARS ORF3a) are similar in their critical protein domains such that they all have 3 transmembrane (TM) regions (Figures 1B, 3A,B) in clockwise arrangement that span across the membrane and connect to the cytosol through a turn-helix-turn (Kern et al., 2021; Figures 3A,B). However, these two proteins only share 73% sequence homology with SARS-CoV ORF3a, missing one amino acid (a.a.) at position 241 (E241). Therefore, SARS-CoV-2 ORF3a is largely unique. Indeed, SARS-CoV-2 ORF3a protein has a novel three-dimensional (3D) structure that shares no homology to any other proteins (McClenaghan et al., 2020; Kern et al., 2021).

SARS-CoV-2 ORF3a protein is produced through viral RTC within DMVs that are generated by fusion of ER membranes (Brant et al., 2021). After ORF3a protein is produced, it is exported from ER to the Golgi apparatus, where it undergoes post-translational modification of O-glycosylation before being inserted into the plasma membrane (Nishimura and Balch, 1997; Oostra et al., 2006). The nucleotide sequence of *ORF3a* has a total of 825 base pairs (bp) that encodes a protein of 275 a.a. with a calculated molecular weight of 31 kilodalton (kD). It has an N-terminal ectodomain (a.a. 1–39), three transmembrane domains (TM1–TM3; a.a. 40–128) spanning a turn-helix-turn, a short cytoplasmic loop (a.a. 175–180) with 8 β-sheets (β1–β8; a.a. 145–235) and a C-terminus (a.a. 239–275). ORF3a protein has a number of well-conserved functional motifs (Figure 1B) that are presumably responsible for its multifunctionalities, including ion channel activity, viral replication, and cytopathogenic effects that link to COVID-19 (Issa et al., 2020; Kern et al., 2021). Specifically, it has a signal peptide (a.a. 1–13), a TNF receptor-associated factor 3 (TRAF3)-binding motif (a.a. 36–40) that associates with the activation of NF-κB and NLRP3 inflammasomes (Siu et al., 2019; Jin et al., 2021); a cysteine-rich domain (a.a. 81–160), in which the C133 residue is critical for maintaining ORF3a homodimerization (Lu et al., 2006; Kern et al., 2021); and a peptide (a.a. 91–133) that connects TM2 and TM3, which are responsible for ion channel activity (Caillet-Saguy et al., 2021). Three caveolin-binding motifs (a.a. 69–77, 107–114, and 141–149) that regulate ORF3a trafficking to the plasma membrane, endosomes, and lysosomes (Padhan et al., 2007). Both the tyrosine-based sorting YXXΦ motif (a.a. 160–163) and the diacidic (S/EGD) motif (a.a. 175–180) are required for protein sorting and transporting ORF3a from the Golgi to plasma membranes (Tan et al., 2004; Minakshi and Padhan, 2014). Finally, ORF3a has a PDZ-binding motif (PBM) at the C-terminal end (SVPL; a.a. 272–275) that could in principle bind numerous host cellular PDZ-containing proteins, suggesting it may be responsible for interaction of ORF3a with a wide range of host cellular functions (Castano-Rodriguez et al., 2018; Caillet-Saguy et al., 2021).

ORF3a protein presents as a homo-tetramer in a dimer-of-dimer configuration (Kern et al., 2021; Figure 3). The protein resides on the plasma membrane and endomembranes including endosomes, lysosomes, Golgi, and ER. The protein monomer (Figures 3A,B) has its N-terminus on the extracellular side. Three TM regions span the membrane and connect to the cytosol through a turn-helix-turn motif after the TM3, which connects to the C-terminus on the cytosolic side of the membrane with 8 sandwich-shaped β-sheets (Figures 3A,B). In a homodimer of the protein (Figure 3C), the 8 β-sheets in the cytosol form 4 pairs of antiparallel β-sheets in opposite orientation that creates an outer face of the C-terminal protein containing β1/β2/β6 and N-terminal half of β7; the inner face of the protein is formed by β3/β4/β5/β8 and C-terminal half of β7. The extracellular N-termini form side openings facing outward (Figure 3C), and two inner faces connect to each other in the cytosol, forming a large hydrophobic inner cavity that is potentially important for ion channel activity and interaction with host cellular proteins (McClenaghan et al., 2020; Kern et al., 2021). In SARS-CoV-2, dimerization of ORF3a may require the presence of C133, which is located in the cysteine-rich pocket near the interface of two oligomers, as a C133A mutation in SARS-CoV ORF3a resulted in loss of oligomerization (Lu et al., 2006). Two other cysteine residues (C148 and C157) may also affect oligomerization, as they are near C133 and close to each other, forming a disulfide bond (McClenaghan et al., 2020). A D142 residue at the top of the short α-helix connecting the transmembrane and cytoplasmic domain provides a single negatively charged pore-lining residue between the inner cavity and the side openings, which suggests that the side opening at the subunit interface may be the path for water and ion movements (McClenaghan et al., 2020; Kern et al., 2021). Note that G187 and G188 are the only two highly conserved glycine resides that separate the two antiparallel β4 and β5 sheets, which are also at the interface of two homo-monomers (Kern et al., 2021). Although the biologic significance of these two resides is currently unknown, they could potentially be important for ORF3a activity during interaction with host cellular proteins. Indeed, a recent study showed that deletion of the residue G188 (∆G188) significantly alters ORF3a-induced cellular oxidative stress and pro-inflammatory responses, leading to much enhanced apoptosis and necrosis (Zhang et al., 2022). Finally, a protein tetramer of ORF3a is joined together by the interface of two homodimers through TM3 (Figure 3D), the outer faces of the neighboring β-sheets, and the hydrophobic cores forming an inner cavity of the protein (McClenaghan et al., 2020; Kern et al., 2021). Overall, it is surmised that ion channel permeation pathways could reside in each dimer and a network of ordered water or ion molecule movement could start from extracellular side openings, passing through the inner cavity and the cytoplasmic domain (McClenaghan et al., 2020; Kern et al., 2021).

## ORF3a as a Viroporin and Its Role in Viral Pathogenesis and COVID-19

### ORF3a as a Viroporin

Viroporin is a viral transmembrane protein that demonstrates ion channel properties in cell membranes. It is typically a hydrophobic protein that oligomerizes in cell membrane and forms ions and small molecules permeable hydrophilic pores (Nieva et al., 2012). The ion channel activities affected by a viroporin could include alteration of cell membrane permeability, Ca^2+^ homeostasis, and membrane remodeling. Thus, the main function of a viroporin affects virion morphogenesis, viral entry, viral replication, and virus release (egress; Nieva et al., 2012). Since both SARS ORF3a are transmembrane proteins with similar structures (Figure 2A) and SARS-CoV ORF3a is a viroporin (Lu et al., 2006; Castano-Rodriguez et al., 2018), it was predicted that SARS-CoV-2 ORF3a might also be a viroporin.

SARS-CoV ORF3a was first shown to have activity of a selective K^+^ channel in a Xenopus oocyte system that was injected with ORF3a (Lu et al., 2006) or in an ORF3a-transfected HEK293 cell line (Muthumani et al., 2009). Mutagenesis studies showed that TM2 and TM3 are needed for the observed ion channel activities (Castano-Rodriguez et al., 2018). This ion channel is inhibited by a K^+^ channel inhibitor barium (Ba^2+^; Lu et al., 2006). In addition, phytochemicals isolated from Chinese herbs, Emodin or Juglanin also inhibit ORF3a-mediated ion channel activity (Lu et al., 2006; Schwarz et al., 2011). Different from SARS-CoV ORF3a, SARS-CoV-2 ORF3a forms a non-selective calcium (Ca^2+^) permeable cation channel in a liposome system (Kern et al., 2021). The channel is permeable to NMDG^+^ or YO-PRO-1, both are large cations, in a way that is reminiscent of other Ca^2+^-permeable channels including TRPV1, TRPA1, and P2X7. Reversal potential shifts in bi-ionic conditions predict permeability ratios (PX/PK^+^) in the order of Ca^2+^ ~ 2 > K^+^ ~ 1 > Na^+^ ~ 0.6 > NMDG^+^ ~ 0.3 (Kern et al., 2021). A single a.a. change (Q57E) within TM1 at top of the cavity, or double mutations (S58L/Q116L) at the base of the TM2–TM3 grooves reduce Ca^2+^ and NMDG^+^ permeability without altering its Na^+^ or K^+^ permeability (Kern et al., 2021; Figure 3C). These data indicate that SARS-CoV-2 ORF3a may act as a non-selective cationic channel with a large pore and high single-channel conductance. Consistent with this notion, non-selective cation channel inhibitors, ruthenium red or polyamine spermidine, block SARS-CoV-2 ORF3a-mediated ion conductance with IC_50_ of 90 ± 10 μM or 10 mM, respectively, in a manner that is unique from those of other known channels (Kern et al., 2021).

Interestingly, neither emodin nor Ba^2+^ inhibits SARS-CoV-2 ORF3a-mediated ion channel activity in the liposome system (Kern et al., 2021). The observed differences in ion channel properties between the two SARS ORF3a proteins suggest a different mode of action of SARS-CoV-2 ORF3a from that of SARS-CoV (Kern et al., 2021). Since different experimental systems were used in those studies, the observed differences could also be due to the systematic difference. Nevertheless, it is clear that SARS-CoV-2 ORF3a is indeed a viroporin that has ion channel properties. However, exactly how SARS-CoV-2 ORF3a functions as an ion channel remains elusive. For more comprehensive reviews of ion channel activities and the differences between SARS-CoV ORF3a and SARS-CoV-2 ORF3a, see (McClenaghan et al., 2020; Gargan and Stevenson, 2021).

### A Role of ORF3a-Mediated Ion Channel Activity in Viral Release

SARS-CoV-2 ORF3a promotes viral release through the lysosomal exocytosis pathway (Chen et al., 2021; Figure 2D). It mediates trafficking of lysosomes to plasma membrane and exocytosis-related SNARE vesicle fusion proteins by facilitating lysosomal targeting of the BORC-ARL8b complex. SARS-CoV-2 ORF3a-mediated lysosomal exocytosis requires activity of the Ca^2+^ channel TRPML3, as elevated cytosolic Ca^2+^ concentration was observed in *ORF3a*-expressing cells but not in control cells, and TRPML3 knockdown blocked ORF3a-mediated lysosomal exocytosis (Chen et al., 2021). The connection between ORF3a-mediated ion channel activity and viral release has also been observed in SARS-CoV (Lu et al., 2006; Schwarz et al., 2011). Inhibition of ORF3a ion channel activity by siRNA in SARS-CoV-infected monkey epithelial FRhK-4 cells reduces the yield of virus production (Lu et al., 2006). In addition, Emodin inhibits ORF3a-mediated ion channel activity and prevents viral release in hCoV-OC43-infected rhabdomyosarcoma RD cells (Schwarz et al., 2011). However, SARS-CoV ORF3a may promote viral release through a different mechanism than SARS-CoV-2 because it does not go through the lysosomal exocytosis pathway (Chen et al., 2021). Interestingly, mutational analysis of SARS-CoV-2 ORF3a shows that residues S171 and W193 are critical for promoting lysosomal exocytosis (Figure 3C). When these two residues (S171 and W193) are introduced into SARS-CoV ORF3a, it gains the ability to promote lysosomal exocytosis (Chen et al., 2021). In addition, adding SARS-CoV-2 ORF3a to CoV-MHV-A59 that has no ORF3a significantly increases virus release (Chen et al., 2021). These data suggest that although ORF3a from other β-CoVs are different from that of SARS-CoV-2, they may share a similar mechanism to promote viral release. Indeed, ORF3a from SARS-CoV, MERS-CoV and SARS-CoV-2 all target lysosomes by disrupting lysosomal acidification to facilitate virus release (Yue et al., 2018; Ghosh et al., 2020). Viruses deficient in ORF3a attenuate their abilities to release viral particles efficiently (Lu et al., 2006; Castano-Rodriguez et al., 2018; Yue et al., 2018; Ghosh et al., 2020).

### ORF3a-Mediated Ion Channel Activity Implicated in Apoptosis and Necrosis

ORF3a induces cell death through both programmed cell death and necrosis (Freundt et al., 2010; Ren et al., 2020). These activities are associated at least in part with ORF3a-mediated ion channel function. For example, inhibition of SARS-CoV ORF3a-mediated K^+^ channel activity by treating ORF3a-producing HEK293 or Vero E6 cells with the K^+^ channel inhibitors, 4-aminopyridine (4-AP) or Ba^2+^, significantly reduced ORF3a-induced caspase-dependent apoptosis (Muthumani et al., 2009). Introduction of triple C127S/C130S/C133S mutations that abolish ion channel activity and interrupt tetramerization of SARS ORF3a (Lu et al., 2006; McClenaghan et al., 2020; Kern et al., 2021; Figures 3C,D), also significantly reduced the level of apoptosis. However, those ion channel inhibitors were unable to block apoptosis completely, suggesting ORF3a-induced apoptosis is only partly associated with ion channel activity (Muthumani et al., 2009; McClenaghan et al., 2020).

ORF3a also induces necrotic cell death that is concurrent with the activation of NLRP3 inflammasome and pro-inflammatory cytokine IL-8 production (Yue et al., 2018; Siu et al., 2019). In one study, SARS-CoV ORF3a induces caspase-1 mediated pyroptosis, a lytic form of necrosis, by interacting with RIP3 (Receptor Interacting Protein 3) that promotes ORF3a oligomerization. To test the potential involvement of ORF3a-mediated ion channel activity in inflammasome activation and cell death, NEK7, an essential mediator of NLRP3 activation downstream of K^+^ efflux, was knocked down in the presence of ORF3a. As result, caspase-1 mediated pyroptosis was reduced, confirming ORF3a acts as a K^+^ channel (Lu et al., 2006) upstream of NEK7 (Yue et al., 2018), and ORF3a is tied to necrosis. However, in a different study, the same triple C127S/C130S/C133S mutations that reduce apoptosis and interfere with K^+^ channel activity did not have major impact on ORF3a-induced pro-inflammatory IL-8 production, which presumably contributes to necrosis. Hence, it was suggested that ORF3a-mediated ion channel activity may not be critical for triggering necrosis (Muthumani et al., 2009; Siu et al., 2019; McClenaghan et al., 2020).

Note that SARS ORF3a induces apoptosis and necrosis in a wide range of eukaryotic cells, including fission yeast (Zhang et al., 2022), fruit flies (Wong et al., 2005; Yang et al., 2020), and various types of human cells, suggesting ORF3a-induced apoptosis and necrosis are highly conserved activities. For instance, SARS-CoV-2 ORF3a induces cellular oxidative stress-mediated cell death in both fission yeast and mammalian cells (Zhang et al., 2022). Both SARS ORF3a induce caspase-8/9-dependent apoptosis in transgenic *Drosophila* (Wong et al., 2005; Yang et al., 2020). SARS-CoV *ORF3a*-expressing fruit flies that were fed the K^+^ channel inhibitor Ba^2+^ partially reduced apoptosis (Chan et al., 2008), consistent with the findings in mammalian cells. In a SARS-CoV-2 ORF3a transgenic model, ORF3a adversely affects longevity and motor function of fruit flies by inducing apoptosis and inflammation in the central nervous system (CNS), suggesting SARS-CoV-2 ORF3a might contribute toward the symptoms of post-COVID conditions in CNS (Yang et al., 2020). Interestingly, fruit flies fed a lysosome deacidification inhibitor chloroquine phosphate (CQ) not only had a prolonged life span but also had reduced cleavage of ORF3a-induced caspase-3 in the CNS (Yang et al., 2020). These data suggest that besides ion channel activity, other cellular events mediated by ORF3a, such as association with endosomes/lysosomes (Ghosh et al., 2020) or activation of cellular innate or pro-inflammatory responses (Zhang et al., 2022), may also contribute to apoptosis and necrosis.

## Roles of SARS-CoV-2 ORF3a in Viral Life Cycle

SARS-CoV ORF3a is a virion-associated protein (Ito et al., 2005; Shen et al., 2005; Huang et al., 2006), as it incorporates into virus-like particles in insect cells co-infected with recombinant baculovirus expressing SARS-CoV ORF3a, E, and M (Shen et al., 2005). It was also found in the virus particles of SARS-CoV-infected Caco2 cells (Ito et al., 2005). Co-immunoprecipitation experiments showed that it interacts specifically with S, M, and E structural proteins (Tan et al., 2004; Zeng et al., 2004; Shen et al., 2005). ORF3a protein can also be released from infected cells as part of SARS-CoV virus particles (Huang et al., 2006). However, no report has yet shown whether SARS-CoV-2 ORF3a is a virion-associated protein. Thus, it would be very interesting to test whether SARS-CoV-2 ORF3a is included in virion. One of the well-accepted methods to test this possibility is to subject purified SARS-CoV-2 viral particles to chromatography, by which it can precisely detect whether ORF3a protein is associated with virion (Shen et al., 2005; de Camargo et al., 2022). Other confirmed or possible roles of SARS-CoV-2 ORF3a in the SARS-CoV-2 viral life cycle are illustrated in Figure 2.

### ORF3a Promotes Viral Entry and Release

Besides virus release promoted by SARS ORF3a (Yue et al., 2018; Ghosh et al., 2020; Chen et al., 2021), ORF3a may also promote viral entry (Figure 2A). One indication is that SARS ORF3a protein localizes on plasma membranes and endomembranes in both transfected and infected cells, which include early endosomes, late endosomes, or lysosomes, as shown by immunostaining for Rab5, Rab7, or LAMP-1, respectively, consistent with ORF3a being present during the entire endocytic pathway (Tan et al., 2004; Padhan et al., 2007; Castano-Rodriguez et al., 2018; Zhang et al., 2020; Figure 2B). In addition, SARS-CoV ORF3a promotes protein internalization, as myc-ORF3a binds and internalizes anti-myc antibody from culture medium into Vero E6 and HeLa cells (Tan et al., 2004), indicating an endocytic process. As the YXXΦ motif of ORF3a is linked to rapid protein internalization (Minakshi and Padhan, 2014), deletion of the cytoplasmic domain of ORF3a, which contains YXXΦ and diacidic motifs (Figure 1B), abolished its movement to the plasma membrane when produced intracellularly (Minakshi and Padhan, 2014). In ORF3a-transgenic *Drosophila*, EGFP-tagged ORF3a was used to test whether the ORF3a function is related to endocytosis by using the “rough eye phenotype” as an indicator (Chang et al., 2004; Wong et al., 2005). The effect of EGFP-ORF3a on the “rough eye phenotype” was tested in an *Eps15* mutant background because Eps15 is required for internalization of TfR. Eps15 is also a YXXΦ-containing protein and an endocytic protein involved in clathrin-mediated endocytosis. The test results support the idea that ORF3a is functionally related to clathrin-mediated endocytosis (Wong et al., 2005).

### Possible Role of ORF3a in Viral Replication and Transcription

A unique feature of the β-CoV is that viral replication and transcription of sgRNAs take place in the RTC on DMVs, which are formed by linking plasma membrane with ER (Angelini et al., 2013; Hagemeijer et al., 2014; Oudshoorn et al., 2017; Figure 2C). Since ORF3a localizes on plasma membrane (Tan et al., 2004; Yuan et al., 2005; Chan et al., 2009) and ER (Yuan et al., 2005), conceivably, ORF3a could also reside on the DMV and participate in viral replication and transcription taking place in the RTC. Electron microscopic observation of infected Vero cells showed that SARS-CoV ORF3a accumulates within vesicles and promotes endomembrane rearrangement and vesicle formation, a prominent clinical feature of SARS-CoV-infected cells from SARS patients (Freundt et al., 2010). To determine the significance of the SARS-CoV accessory proteins in viral replication *in vitro* and in mice, five of the 8 accessory SARS-CoV ORFs were deleted including ORF3a, OF3b, ORF6, ORF7a, and ORF7b (Yount et al., 2005). Although none of individual or combination deletions dramatically influenced replication efficiency in cell culture or the levels of viral RNA synthesis, ORF3a deletion showed the greatest reduction in virus growth (Yount et al., 2005). By using a similar strategy in a different study, K18-ACE2 transgenic mice, which express human ACE2 under the control of a human K18 promoter, was used to determine the significance of the SARS-CoV-2 accessory proteins (Silvas et al., 2021). Because K18-hACE2 are susceptible to SARS-CoV-2, and K18 promoter directs hACE2 expression to epithelia, including airway epithelia where infection typically begins, this is a useful animal model to study viral pathogenesis of SARS-CoV-2. As results, K18 hACE2 transgenic mice infected with virus carrying a *ORF3a* deletion showed less mortality, lower lung viral titers, and less tissue damage, indicating an important role for ORF3a in viral pathogenesis and COVID-19 (Silvas et al., 2021). Considering that SARS ORF3a is necessary and sufficient for SARS-CoV-induced endomembrane rearrangement and vesicle formation (Freundt et al., 2010), it is likely that SARS ORF3a may play a functional role on DMV in viral transcription and replication. This possibility needs to be further evaluated.

### A Prominent Role of ORF3a in Virus Reproduction

ORF3a is an accessory protein, that is, the virus does not depend on it for its reproduction. However, deletion or transcriptional knockdown of *ORF3a* from the SARS-CoV genome results in significant reduction of virus growth (Yount et al., 2005; Akerstrom et al., 2007; Silvas et al., 2021). Importantly, the presence of ORF3a is essential for viral reproduction when E protein is absent, as SARS-CoV and SARS-CoV-2 missing both E and 3a proteins are not viable in Vero E6 cells or infected BALB/c mice (Castano-Rodriguez et al., 2018; Zhang et al., 2021). Thus, ORF3a and E are required for maximizing virus reproduction. Comparatively, however, E protein appears to be more important than ORF3a in both viral replication and virulence because loss of E dramatically decreased the virus production and abolished viral pathogenesis; while the virus with deletion of ORF3a merely caused weight loss in mice (Castano-Rodriguez et al., 2018).

A common feature between ORF3a and E is that they both contain a PDZ (PSD-95/Dlg/ZO-1)-binding motif (PBM), which in principle binds to hundreds of host cellular PDZ-containing proteins, and thereby affect a wide range of host cellular functions (Castano-Rodriguez et al., 2018). The PDZ domain is a stretch of 80–90 a.a. that is commonly found in signaling proteins (Javier and Rice, 2011). Viral proteins with PBMs often bind to cellular PDZ-containing proteins (Javier and Rice, 2011) to regulate signaling complexes at cellular membranes (Lee and Zheng, 2010), to control tight junction formation, cell polarity establishment, or to induce apoptosis (Javier and Rice, 2011). The PBM sequence consists of 4 a.a. at the C-terminal end of a protein, with the last residue always being hydrophobic (Castano-Rodriguez et al., 2018). The PBM residues of SARS-CoV-2 ORF3a are SVPL, which is highly conserved among all sarbecoviruses (Caillet-Saguy et al., 2021; Figure 1B). In a high-throughput holdup assay that measures the binding affinity of a PBM-carrying viral protein to PDZ-containing cellular proteins, SARS-CoV-2 E and ORF3a were used as bait against the entire human PDZome (Belotti et al., 2013). Eight PDZ-containing cellular proteins (TJP1, NHERF4, NHERF3_4, RGS3, PARD3B, PARD3, FRMPD4, and NHERF3) showed significant interactions with ORF3a. Among them, 2 proteins (TJP1 and PARD3) bind to both ORF3a and E proteins (Caillet-Saguy et al., 2021). siRNA knockdown of two PDZ-containing cellular proteins such as *PARD3* and *RSG3* in lung epithelial A549 cells showed significant decrease of SARS-CoV-2 replication. Conversely, silencing of *PARD3B* increased virus replication (Caillet-Saguy et al., 2021). PARD3 is involved in the formation of adherens and tight junctions. It binds to a transcription factor YAP (Yes-associated protein) that regulates the activation of the Hippo pathway, a signaling pathway modulating cell proliferation and cell death (Yu and Guan, 2013). RGS3 (G protein signaling 3) is largely a cytosolic protein and a member of the regulator of G protein signaling (RGS) family. It is a GTPase-activating protein that inhibits G protein-mediated signal transduction, but G protein activation leads to translocation of RGS3 to the plasma membrane (Lu et al., 2001). Although the specific mechanism involving PARD3 and RGS3 in ORF3a/E-mediated viral reproduction is currently unknown, it would be interesting to interrupt the interactions of ORF3a/E with these PDZ-containing proteins and to determine whether they would reduce virus reproduction.

Based on the necessary role of ORF3a and E proteins in viral reproduction and virulence, a single-round SARS-CoV-2 infection system was established to recapitulates authentic viral replication without major concern of viral virulence and biosafety (Zhang et al., 2021). To achieve this goal, both ORF3a and E genes were deleted from the viral genome. Instead, a Vero E6 cell line producing the ORF3a and E proteins under a doxycycline inducible promoter was used to provide these two proteins *in-trans* in such a way that the generated virions would infect naive cells for only one round, and would not produce wild-type SARS-CoV-2 due to the lack of ORF3a and E proteins (Zhang et al., 2021).

## ORF3a–Host Interaction and Its CLINICAL Consequences

Increasing evidence suggests that ORF3a protein plays an important role in viral pathogenesis and contributes to the severity of SARS and COVID-19. For example, many convalescent SARS patients develop antibodies against ORF3a (Liu et al., 2004; Zhong et al., 2006), and sera from COVID-19 patients also show high levels of IgG and IgA reactivities specifically to ORF3a in addition to structural proteins (Camerini et al., 2021). High titers of anti-ORF3a antibody were also found in SARS-CoV-2-infected patients, suggesting active ORF3a-host interaction with clinical consequences (Hachim et al., 2020).

### Host Cellular Innate Immune Response

**Figure 4:**
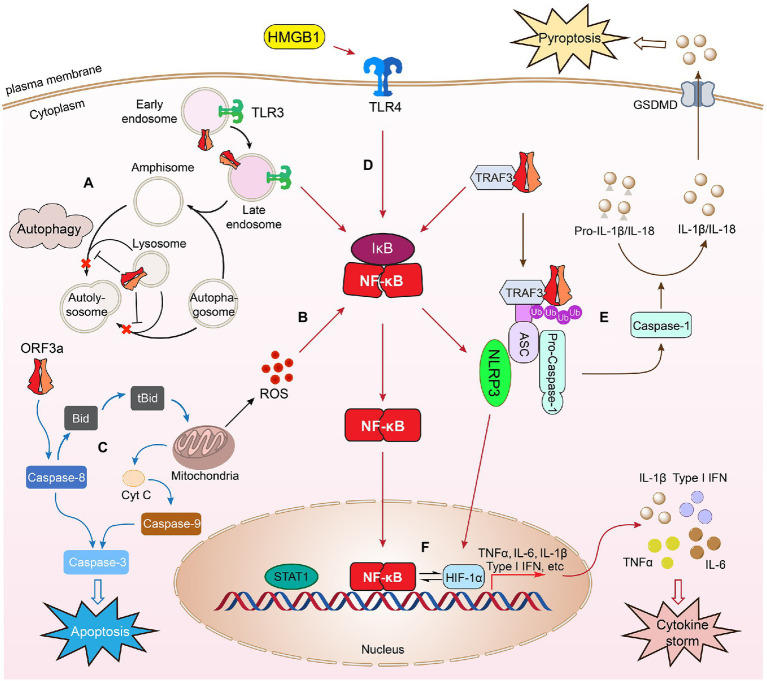
SARS-CoV-2 ORF3a–host cell interaction. Expression of SARS-CoV-2 ORF3a in infected cells triggers cellular innate immune responses such as autophagy **(A)**. Autophagy is an important cellular antiviral response to trap viruses in autophagosomes for lysosomal degradation. An autophagosome with trapped virus fuses with an endosome to generate an amphisome, which then fuses with lysosomes, forming an autolysosome, where virus cargos can be recycled by lysosomal enzymes. ORF3a counteracts cellular autophagy activity by blocking fusion of autophagosomes/amphisomes with lysosomes. ORF3a also triggers cellular oxidative stress response leading to increased ROS production **(B)** and induces mitochondria-dependent and mitochondria-independent apoptosis **(C)**. SARS-CoV-2 ORF3a induces cellular pro-inflammatory immune responses to activate cytokine (TNFα, IL-6, and IFN-1β) production through NF-κB, TLR4, or TLR3-mediated process **(D)**. ORF3a interacts with TRAF3 triggering downstream NF-κB activation and cytokine induction leading to activation of NLRP3 inflammasomes. Formation of the NLRP3 inflammasome activates caspase-1 that converts pro-IL-1β to IL-1β through a proteolytic processing leading to pyroptosis **(E)**, which is an inflammatory form of lytic cell death that promotes rapid clearance of invading viruses and enhances host antiviral response (Jorgensen and Miao, 2015). ORF3a-induced NF-κB activation could activate NLRP3 inflammasomes, which in turn activates HMGB1 and HIF-1α to promote the production of pro-inflammatory cytokines and possibly induction of cytokine storm **(F)**. As a result, it induces cell death through apoptosis, necrosis, and pyroptosis, leading to tissue damage, COVID-19 or post-COVID conditions. This figure is generated using Adobe Illustrator 2020.

SARS ORF3a protein triggers a range of host cellular innate immune responses, including cellular stress responses such as autophagy in infected cells. Autophagy is a normal cellular process that maintains cellular homeostasis, but autophagy can also be activated as an important cellular antiviral response to trap viruses in autophagosomes for their lysosomal degradation (Figure 4A; Choi et al., 2018). An autophagosome trapped with viruses will fuse with endosome to generate an amphisome, which then fuses with lysosomes, forming an autolysosome, where cargo with trapped viruses will be recycled by lysosomal enzymes (Corona and Jackson, 2018). Fusion of autophagosomes/amphisomes with lysosomes is a highly regulated process that requires the assembly of HOPS and SNARE protein complexes during cellular innate autophagic response to viral infection (Corona and Jackson, 2018).

To counteract host cellular antiviral autophagic response, SARS ORF3a blocks the fusion of autophagosomes/amphisomes with lysosomes (Koepke et al., 2021; Miao et al., 2021), or highjacks autophagosomes to facilitate the formation of DMVs for its own viral transcription and replication on RTC (Fung and Liu, 2019; Gassen et al., 2019). Both SARS ORF3a proteins localize on endosomes/lysosomes (Padhan et al., 2007; Castano-Rodriguez et al., 2018; Zhang et al., 2020; Miao et al., 2021). SARS-CoV-2 ORF3a counteracts cellular autophagy activity by causing lysosomal damage and impairing its function (Miao et al., 2021), thus blocking fusion of autophagosomes/amphisomes with lysosomes (Koepke et al., 2021; Miao et al., 2021). Specifically, SARS-CoV-2 ORF3a sitting on endosomes blocks autolysosome formation through direct interaction with VPS39, a component of HOPS, to prevent the interaction of HOPS with a SNARE protein STX17 (Miao et al., 2021). The same counteract-autophagy activity by ORF3a was also demonstrated in a different study, where it was found that ORF3a interacts with VPS39 and prevents the binding of HOPS through a different protein, Rab7 (Zhang et al., 2021). Results of these two studies suggest that ORF3a blocks the fusion of autophagosomes/amphisomes with lysosomes through interactions possibly with a group of proteins, rather than an individual protein. Indeed, SARS-CoV-2 ORF3a also blocks autophagy by interacting with Beclin 1, an essential scaffold autophagy protein, to differentially modulate two Beclin-associated protein complexes, PI3KC3-C1 (Beclin-1-Vps34-Atg14) and PI3KC3-C2 (Beclin-1-Vps34-UVRAG; Qu et al., 2021). These results suggest a similar counteracting effect of SARS ORF3a protein on the host autophagic response. In addition, SARS ORF3a could also evade host cellular autophagy by hijacking autophagosomes in order to facilitate the formation of DMVs for its own viral transcription and replication on RTC (Fung and Liu, 2019; Gassen et al., 2019). For instance, SARS-CoV ORF3a triggers autophagy during endocytosis that promotes the formation of DMV and viral replication (Yang and Shen, 2020). Expression of SARS-CoV-2 ORF3a significantly increases SARS-CoV-2 replication in infected Calu-3 cells with an elevated autophagic response. Genetic abrogation of Atg3 and Atg5, two essential proteins for autophagosome formation, markedly reduced viral replication (Qu et al., 2021), indicating SARS-CoV-2 ORF3a facilitates viral replication through counteraction against host cellular autophagic responses.

SARS-CoV-2 ORF3a also elicits host cellular oxidative stress that contributes to ORF3a-induced apoptosis and necrosis (Zhang et al., 2022). Increased oxidative stress is another common cellular antiviral response to viral infection (Fernandes et al., 2020). Sustained viral infection leads to the accumulation of reactive oxygen species (ROS) in infected cells or tissues that could lead to cell death and tissue or organ damage (Li et al., 2017; Holze et al., 2018). Increasing evidence suggests that excessive ROS production is a major cause of local or systemic tissue damage and contributes to the severity of COVID-19 (Laforge et al., 2020; Kozlov et al., 2021). SARS-CoV-2 ORF3a-induced cellular oxidative stress and cell death appears to be a highly conserved activities, since elevated production of ROS and cell death are observed in both fission yeast and human A549 and 293 T cells (Zhang et al., 2022). However, the underlying molecular mechanism of SARS-CoV-2 ORF3a-induced ROS production and associated cell death are currently unknown. It is known, however, that SARS-CoV-2 ORF3a induces cell death through mitochondrial damage and mitochondria-mediated ROS production that facilitates SARS-CoV-2 infection and pro-inflammatory cytokines production (Tan et al., 2021; Figure 4B). Consistently, mitochondria-released ROS production and ion channel K^+^ efflux are required for the activation of NLRP3 inflammasome in lipopolysaccharide-primed macrophages and caspase-1 induced pyroptosis (Chen et al., 2019). Therefore, it is likely that ORF3a-induced cell death is at least in part mediated through mitochondria-mediated ROS production. Apoptosis induced by SARS ORF3a proteins are also mediated through mitochondria-dependent (intrinsic) and mitochondria-independent (extrinsic) pathways (Law et al., 2005; Waye et al., 2005; Padhan et al., 2007; Freundt et al., 2010; Ren et al., 2020; Zhang et al., 2021, 2022; Figure 4C). For example, SARS-CoV ORF3a activates caspase-8 *via* Bid to tBid conversion (Law et al., 2005), caspase-9 increase, and cytochrome c release from mitochondria (Padhan et al., 2008). In addition, Bax, p53, and p38 MAP kinase also play roles in ORF3a-induced apoptosis (Padhan et al., 2008). SARS-CoV-2 ORF3a-induced apoptosis is mediated through similar mode of action to SARS-CoV ORF3a, that is, it induces apoptosis through the cleavage of Bid, caspase-8, and caspase-9 or the release of cytochrome c (Ren et al., 2020) leading to caspase-3 cleavage (Ren et al., 2020; Zhang et al., 2022). Two ORF3a mutants, ORF3a-CS (C130S/C133S) and ORF3a-YA (Y160), which interrupt the cysteine-rich motif (a.a. 81–160) and the tyrosine-based sorting motif (YXXΦ; a.a.160–163), and reduce the association with the plasma membrane, reduced ORF3a-induced apoptosis. Interestingly, plasma membrane association is required for the pro-apoptotic activity of SARS-CoV-2 ORF3a, but not for SARS-CoV ORF3a, suggesting a subtle difference of these two ORF3a proteins in the induction of apoptosis (Ren et al., 2020).

SARS ORF3a-induced necrosis could also be mediated through different pathways (Freundt et al., 2010; Yue et al., 2018; Siu et al., 2019; Zhang et al., 2022). For example, ORF3a-induced necrosis is associated with Golgi fragmentation and membrane rearrangement (Freundt et al., 2010). SARS-CoV ORF3a induces necrosis by lysosomal damage through interruption of Rip3-mediated oligomerization of ORF3a (Yue et al., 2018). In addition, SARS-CoV ORF3a-induced necrosis is accompanied by the secretion of pro-inflammatory cytokines (Yue et al., 2018; Siu et al., 2019). SARS-CoV-2 ORF3a-induced apoptosis and necrosis also correlate with the activation of pro-inflammatory cytokine production and possibly link it to the induction of oxidative stress (Zhang et al., 2022). Additional study is needed to confirm these mechanisms.

Overall, ORF3a elicits host cellular innate immune responses that lead to apoptosis and necrosis. This cell death-inducing capacity appears to be a highly conserved cellular response among eukaryotic cells including fission yeast (Zhang et al., 2022), *Drosophila* (Wong et al., 2005; Yang et al., 2020), and mammalian cells (Ren et al., 2020; Zhang et al., 2022), suggesting ORF3a impinges upon fundamental cell death pathways.

### Host Cellular Pro-inflammatory Immune Response

Besides the induction of host cellular innate antiviral immune responses, SARS-CoV-2 infection also triggers a series of host cellular antiviral and pro-inflammatory immune responses that result in the production and release of cytokines and chemokines (Mogensen and Paludan, 2001). The balance between a cellular antiviral immune response and a pro-inflammatory overreaction determines the clinical outcome. In the case of SARS-CoV-2 infection, over-inflammatory responses in patients with COVID-19 could induce the so-called cytokine storm that is a major cause of disease severity and death (Del Valle et al., 2020; Sayah et al., 2021).

The expression of SARS-CoV-2 *ORF3a* in human lung, kidney, heart, and cervical epithelial cells triggers moderate level of NF-κB-mediated cytokine production including TNF-α, IL-1β, IL-6, and IFN-β1 in a time-dependent manner (Su et al., 2021; Zhang et al., 2022). High serum IL-6 and TNF-α levels are two strong and independent survival predictors of patient with COVID-19 (Del Valle et al., 2020; Sayah et al., 2021). In addition, Toll-Like receptors 3 (TLR3) and TLR4 are also elevated by ORF3a (Zhang et al., 2022). Both TLR3 and TLR4 recognize double-stranded RNA and trigger antiviral production of type I IFNs and pro-inflammatory cytokines. TLR4 is a cell membrane receptor (Aboudounya and Heads, 2021) and TLR3 resides on the endosomal membrane in epithelial cells (Matsumoto et al., 2014; Figure 4D). TLR3 induces pro-inflammatory cytokine production through a TLR adaptor molecule, TRIF (Toll/IL-1R domain-containing adaptor inducing IFN; Matsumoto et al., 2014), whereas TLR4-mediated pro-inflammatory cytokine production is primarily through NF-κB. Both TLR3 and TLR4-mediated pro-inflammatory responses contribute to the severity of COVID-19 (Mather et al., 2020; Brandao et al., 2021). TLR4-mediated production of IL-6 and TNF-α is associated with the severity of COVID-19 in patients with cardiometabolic comorbidities (Brandao et al., 2021). Inhibition of TLR3 by famotidine decreases IL-6 (Mukherjee et al., 2021) and reduces the risk of intubation and death in patients hospitalized with COVID-19 and alleviates symptoms in non-hospitalized patients with COVID-19 (Mather et al., 2020). Therefore, it is likely that ORF3a-induced IL-6 and TNF-α production through NF-κB, TLR3 or TLR4 could all contribute to the severity of COVID-19. Additional study is needed to explore these possibilities.

Although moderate levels of NF-κB-mediated cytokines are induced by ORF3a (Su et al., 2021; Zhang et al., 2022), expression of a single a.a. deletion at residue 188 (∆G188) of ORF3a resulted in markedly increase of NF-κB and downstream cytokines of TNF-α, IL-6, and IFN-β1 (Zhang et al., 2022). The G188 residue is one of the two highly conserved glycine resides that separate two antiparallel β4/β5 sheets of the ORF3a homodimers (Kern et al., 2021; Figure 3C). Thus, the ∆G188 deletion may interrupt the configuration of β4/β5 sheets affecting dimerization of the protein and the C-terminal end, where ion channel activity or interaction of ORF3a with host cellular proteins may take place. One possible explanation for the stronger effect of the ∆G188 mutant than wild type is that the wild-type ORF3a protein might be restricted by host cellular protein(s) through direct protein–protein interaction (Caillet-Saguy et al., 2021), as shown in other viral infections (Gaddy and Lyles, 2005). This possibility warrants additional investigation.

SARS-CoV ORF3a activates NF-κB, TNF-α and promotes cytokines and chemokines productions (Kanzawa et al., 2006; Obitsu et al., 2009; Chen et al., 2019). Interestingly, ORF3a induces IL-6 and IL-18 production through activation of the NLRP3 (Nod-like receptor family, pyrin domain-containing 3) inflammasome (Chen et al., 2019), a multimeric protein complex that triggers the secretion of pro-inflammatory cytokines (Jorgensen and Miao, 2015; Yang et al., 2019; Freeman and Swartz, 2020). Clinically, over-regulated or dysregulated NLRP3 inflammasome activation in SARS-CoV and SARS-CoV-2-infected patients could trigger a cytokine storm that causes tissue damage and organ failure in patients with severe SARS or COVID-19 leading to death (Freeman and Swartz, 2020; van den Berg and Te Velde, 2020). SARS-CoV ORF3a-induced activation of the NLRP3 inflammasome requires K^+^ efflux and oxidative stress-induced ROS production, which results in further secretion of IL-1β and induction of pyroptosis (Chen et al., 2019). Pyroptosis is an inflammatory form of necrosis that promotes rapid clearance of invading viruses and enhances host antiviral response (Jorgensen and Miao, 2015; Figure 4E). Specifically, formation of the NLRP3 inflammasome activates caspase-1 that converts pro-IL-1β to IL-1β through a proteolytic processing leading to pyroptosis (Kayagaki et al., 2015; Shi et al., 2015). ORF3a-induced NF-κB expression also promotes activation of caspase-1 and IL-1β maturation through interaction with a ubiquitin ligase TNF receptor-associated factor 3 (TRAF3), which promotes ubiquitination of ASC, an adaptor protein that activates caspase-1 (Siu et al., 2019; Jin et al., 2021). Both SARS ORF3a proteins induce pyroptosis through the activation of caspase-1 and IL-1β maturation (Yue et al., 2018; Siu et al., 2019; Gowda et al., 2021; Figure 4E). These data suggest that the SARS-CoV-2 ORF3a function associated with the activation of NLRP3 inflammasome or a cytokine storm determines the severity of COVID-19 in patients. Although SARS-CoV-2 infection triggers activation of the NLRP3 inflammasome that results in tissue damage and severe COVID-19 (Freeman and Swartz, 2020; van den Berg and Te Velde, 2020), it is unclear at present whether ORF3a is directly involved in activating the NLRP3 inflammasome. The suspicion is that it might, because SARS-CoV-2 ORF3a activates one of the downstream effectors of the NLRP3 inflammasome, HMGB1 (high mobility group box 1), and induces caspase-1-mediated pyroptosis (van den Berg and Te Velde, 2020; Yang et al., 2020; Gowda et al., 2021). HMGB1 is a ubiquitous protein released by microglia or macrophages upon NLRP3 inflammasome activation and promotes TLR4- and receptor for advanced glycation end products (RAGE)-mediated pro-inflammatory cytokine production (Paudel et al., 2018; Peng et al., 2021; Figure 4F). Highly elevated serum levels of HMGB1 are found in COVID-19 patients (Bolay et al., 2021), which correlates with poor prognosis of these patients (Chen et al., 2020). An HMGB1 inhibitor, glycyrrhizin, reduces ORF3a-induced HMGB1 release and production of IL-1β, IL-6, and IL-8 and further prevents ORF3a-induced caspase-1-mediated pyroptosis (Gowda et al., 2021). Furthermore, glycyrrhizin inhibits SARS-CoV-2 replication in Vero E6 cells, suggesting SARS-CoV-2 ORF3a-mediated HMGB1 release is associated with host cellular pro-inflammatory responses and viral replication (Gowda et al., 2021).

Besides activation of the NLRP3 inflammasome and HMGB1, hypoxia-inducible factor-1α (HIF-1α) signaling pathway (Figure 4F) is also elevated in elderly patients with COVID-19 (Tan et al., 2021). The elevated HIF-1α is particularly interesting as it is associated with a hyper-pro-inflammatory response and cytokine production (IL-6, IL-1β, IFN-β, and TNF-α, etc.) with high mortality (Tan et al., 2021). Significantly, one of the major clinical manifestations in patients with severe COVID-19 infection is hypoxia, that is, insufficient oxygen level in affected tissue and blood, and HIF-1α mediates cellular responses to low oxygen concentration (Jahani et al., 2020). Consistent with this, elevated HIF-1α signaling is seen in patients with severe COVID-19 experiencing low levels of oxygen in affected tissues and blood, and HMGB1 activates HIF-1α to promote pro-inflammatory cytokine production *via* NF-κB in activated monocytes under hypoxic condition (Peng et al., 2021; Figure 4F). *In vitro*, SARS-CoV-2 infection induces elevation of HIF-1α and promotes cytokine production (Tian et al., 2021). Moreover, ORF3a promotes HIF-1α production through the ROS production from mitochondria, which in turn facilitates SARS-CoV-2 infection and pro-inflammatory cytokines production (Tian et al., 2021). Overall, these findings provide a possible framework on how SARS-CoV-2 ORF3a induces a cytokine storm in patients with COVID-19 especially under hypoxic conditions. In that situation, we hypothesize that SARS-CoV-2 ORF3a triggers a cytokine storm by activating NLRP3 inflammasomes, which in turn activates HMGB1 and HIF-1α to promote the overproduction of pro-inflammatory cytokines (Figure 4).

## Natural ORF3a Variants and Potential Association With Viral Pathogenesis and COVID-19

### Origin and Evolution

Among the seven hCoVs, ORF3a only presents in SARS-CoV and SARS-CoV-2 (Kern et al., 2021), implicating its unique involvement in SARS viruses. Thus, ORF3a protein must have been acquired recently during viral evolution by SARS viruses from the CoV lineage. This is evident because no ORF3a homologues are detected even in its close relatives of embercovirus, a subgenus of β-CoV, γ-CoV, or δ-CoV that includes hCoV-HKU1 and hCoV-OC43 (Ouzounis, 2020; Kern et al., 2021; Tan et al., 2021). Protein sequence comparisons and computer protein structure modeling analyses suggest that ORF3a might be derived originally from M gene in the CoV lineage (Ouzounis, 2020; Tan et al., 2021). Using SARS-CoV-2 ORF3a protein structure as a template to search for possible structural homologues, several families of viral proteins were revealed including SARS-CoV M, ORF5 from MERS-CoVs, ORF3c from β-CoVs, and ORF3b from α-CoVs (Tan et al., 2021). All these structural homologues share a common 3-TM region followed by a β-sandwich domain. In addition, they show unique polar residues in the inner cavity of the proteins (Tan et al., 2021). The host range of those viruses might also provide a hint about the source of SARS ORF3a. While no structural homologues of ORF3a are found in CoVs that infect rodents, birds, or pigs, close protein structural homologues of ORF3a are found in CoVs that primarily infects bats, pangolin, and civets (Kern et al., 2021).

Phylogenetic analysis of the SARS-CoV-2 genome and calculation of evolutionary rates of a.a. changes overtime suggest that ORF3a is selected for its diversity and functional adaptation during viral evolution (Velazquez-Salinas et al., 2020; Jungreis et al., 2021; Tan et al., 2021). For instance, the residue 99 of ORF3a is selected for an adenine (A99; Lo Presti et al., 2020), which is highly conserved at the junction of TM2 and TM3 (Figure 3D). The residue 251 is also selected for from glycine to valine (G251V) that resides at a highly conserved region of the cytoplasmic C-terminal end, suggesting this residue might be important for its function (Velazquez-Salinas et al., 2020). Indeed, protein structural analysis predicts that mutations of G251V could significantly affect the overall protein structure of ORF3a (Wu et al., 2021).

There is also an intriguing idea emerging to suggest that SARS ORF3a might co-evolve with S protein as positive selection was observed in ORF3a along with S during the SARS outbreak in 2003 (McClenaghan et al., 2020; Tan et al., 2021). In addition, SARS-CoV ORF3a interacts with S protein (Tan et al., 2004; Zeng et al., 2004; Shen et al., 2005) by forming disulfide bonds (Zeng et al., 2004). Emodin, an ion channel inhibitor of SARS-CoV ORF3a, blocks the interaction of S protein with ACE2 (Ho et al., 2007), indicating that ORF3a may function as a modulator of S protein (Tan, 2005). Although no report has yet shown direct interaction of SARS-CoV-2 ORF3a with S protein, analysis of protein structures and molecular docking predict that SARS-CoV-2 ORF3a may interact with S protein. It was further predicted that Q57H and G251V mutant ORF3a also bind to S protein stronger than the wild-type ORF3a (Wu et al., 2021). Thus, it would be interesting to test whether these SARS-CoV-2 ORF3a proteins indeed bind to S protein and further to access their roles in viral infection.

### Emerging Natural Variants and Potential Association With Viral Pathogenesis and COVID-19

Due to the error-prone nature of RNA viral replication, the SARS-CoV-2 genome mutates with high frequency resulting in continued emergence of new variants to improve its fitness in human infection (Domingo et al., 2021). Continued mutations of S protein are of major concern as they alter the ability of the virus to transmit, infect and cause COVID-19 (Chakraborty et al., 2021; Changrob et al., 2021). Continued emergence of ORF3a protein variants might also be of concern as its mutations could affect its role in viral pathogenesis, severity of COVID-19, and contribution to post-COVID conditions as described above (Majumdar and Niyogi, 2020; Hassan et al., 2021; Nagy et al., 2021). Using the first SARS-CoV-2 virus isolated from Wuhan, China as a reference sequence (Wu et al., 2020), continual monitoring and comparison of genome sequences in the GISAID database collected worldwide since the onset of the pandemic has revealed a large numbers of natural non-synonymous mutations of SARS-CoV-2 ORF3a that span throughout the entire protein (Issa et al., 2020; Azad and Khan, 2021; Bianchi et al., 2021; Nagy et al., 2021). Among those mutations, Q57H and G251V are the two most abundant ORF3a mutations (Issa et al., 2020; Bianchi et al., 2021; Figure 3D). Besides single a.a. changes, double mutants also are found to pair with Q57H or G251V (Issa et al., 2020; Bianchi et al., 2021). Although most of the mutations appear to be random, some a.a. changes could potentially be functionally relevant as they occur at relatively high frequencies in functionally relevant domains (Issa et al., 2020; Bianchi et al., 2021). For instance, among 17 unique ORF3a variants discovered out of 70,752 SARS-CoV-2 ORF3a variants, 10 are in the TM domains that are in contact with central pore or side tunnels of the protein, the other 7 mutations are at the extracellular N-terminus or the C-terminal cytoplasmic β-sheet domains (Bianchi et al., 2021). Nevertheless, whether those natural ORF3a variants affect the function of ORF3a is currently unknown.

One study found that 18 unique a.a. changes in the ORF3a protein are associated with higher mortality in patients with COVID-19 (Majumdar and Niyogi, 2020). This study compared the ORF3a mutational profile with the rate of infection and mortality in over 20,000 COVID-19 positive cases from 23 countries (Majumdar and Niyogi, 2020). They found that some ORF3a mutations were present only in those countries where high infection and high mortality rates were shown, but they were not present in countries with low infection and low mortality rates. It was suspected that those ORF3a mutations could alter ORF3a functions or skip recognition by B cell epitopes (Majumdar and Niyogi, 2020). No experimental data were provided to verify those predictions. However, in a different study, complete loss of T cell responsiveness was attributed to a Q213K mutation in the A∗01:01-restricted CD8+ ORF3a epitope FTSDYYQLY_207-215_ that is part of the β7 sheet (de Silva et al., 2021; Figure 3D).

Unique ORF3a mutations also associate with different levels of COVID-19 severity (Nagy et al., 2021). This study correlated ORF3a mutations that were discovered from a total of 149,061 genome sequences deposited in the GISAID database with a total of 7,702 individuals who were infected with SARS-CoV-2 with various degrees of disease severity. They found that a G196V mutant, which is between β5 and β6 sheets (Figure 3D), is associated with those patients who had mild disease, that is, asymptomatic or not hospitalized; the Q57H and G251V mutants are associated with those patients who were hospitalized in ICU or had severe outcome; and the S253P mutation links to those patients with deadly outcomes (Nagy et al., 2021). Both residues 251 and 253 are at the free C-terminal end, and Q57H is near the N-terminal end within TM1 (Figure 3D). A 4-a.a. deletion (a.a. 11–14) at the N-terminal end of ORF3a was also found to associate with an ICU COVID-19 patient who suffered severe disease (Lednicky et al., 2021). The virus with this ORF3a deletion mutant showed reduced viral replication and cytopathic effects compared with the wild-type control virus. Interestingly, no similar viruses were isolated from this hospital during the same time period, suggesting this mutant virus may have lost its ability to transmit (Lednicky et al., 2021). All these studies suggest that certain ORF3a mutations are indeed correlated with the severity of COVID-19 (Majumdar and Niyogi, 2020; Lednicky et al., 2021; Nagy et al., 2021).

Some ORF3a mutants are found in the virus of interest (VOI) and virus of concern (VOC) as defined by World Health Organization (WHO; Table 1). For instance, the Q57H mutation is present in Epsilon variant, whereas the Q57H/S171L mutations are found in Beta variant (Tzou et al., 2020). While Q57H mutation is in TM1 (Figure 3D), suggesting possible alteration of ORF3a function, a.a. at residue 171 are diverse within β3 sheet (Kern et al., 2021). Interestingly, the emergence of Q57H was reported to associate with the 4^th^ wave of resurgence of SARS-CoV-2 in Hong Kong (Chu et al., 2021). ORF3a protein with the Q57H mutation was predicted to have stronger binding affinity to S protein than the wild-type ORF3a (Wu et al., 2021), and it also associates with disease severity (Nagy et al., 2021). Yet, *in vitro* experimental data show that the activities of Q57H ORF3a are comparable to the wild-type ORF3a in its ability to elicit host cellular innate and pro-inflammatory responses and induce cell death in lung and kidney epithelial cells (Zhang et al., 2022). However, these results cannot rule out the possibility that Q57H may have other activities that are different from wild type. The Gamma variant carries a single S253P alteration or combined S253P/D155Y mutations. Both D155 and S253 residues are highly conserved among sarbecoviruses. D155 is at the junction of β1 and β2 sheets, and S253 is within a stretch of the well-conserved cytoplasmic domain of the C-terminus (Figure 3D). Therefore, mutations of D155Y and/or S253P could potentially alter the ORF3a activities. In contrast to the potentially functionally relevant mutations described above, S26L mutation found in the Delta variant may not have any functional significance, as this residue is quite diverse among sarbecoviruses (Kern et al., 2021).

**Table 1:** Summary of the emerging SARS-CoV-2 ORF3a mutant variants.

Viral variants	Pango* lineage	First detection and location	ORF3a mutations**	Possible mutational effect and reference
**Virus of Interest (VOI)** ***				
Lambda	C.37	Peru, Dec-2020	None	n/a
Mu	B.1.621	Colombia, Jan-2021	Q57H, del256/257	V256/N257 are at a highly conserved region of the C-terminal
Epsilon	B.1.427	California, USA, July 2020	Q57H	It may render stronger binding affinity to S protein than wild-type ORF3a (Wu et al., 2021); also associates with disease severity (Nagy et al., 2021).
**Virus of Concern (VOC)** ***				
Alpha	B.1.1.7	United Kingdom, Sep-2020	None	n/a
Beta	B.1.351	South Africa, May-2020	Q57H, S171L	S171 within β3; see note on Q57H
Gamma	P.1	Brazil, Nov-2020	S253P, D155Y	D155 at junction of β1 and β2 sheets; S253 within a well-conserved C-terminal end
Delta	B.1.617.2	India, Oct-2020	S26L	At the extracellular N-terminal
Omicron	BA.1/B.1.1.529	South Africa Nov-2021	None	n/a (Desingu et al., 2022; Saxena et al., 2022)
	BA.2/B.1.1.529.2	South Africa Nov-2021	T223I	T223I is at junction of β7 and β8 sheets (Desingu et al., 2022; Mahase, 2022)
	BA.3/B.1.1.529.3	South Africa, Nov-2021	T223I	T223I is at junction of β7 and β8 sheets (Desingu et al., 2022)

n/a, not applicable.

* A phylogenetic name of a distinctive viral variant (Rambaut et al., 2020).

** Data are from Stanford University Coronavirus Antiviral and Resistance Database (Tzou et al., 2020).

*** VOI and VOC are defined by WHO.

Trending of viral protein mutation frequencies among SARS-CoV-2 that are circulating worldwide showed an overall decline of mutation frequency of ORF3a over time (Hassan et al., 2021). The significance of this decline in viral pathogenesis and ORF3a evolution is unclear. An intriguing observation is that in contrast to a significant and large number of mutations found in other part of the viral genome of Omicron variants (BA.1, BA.2, and BA.3), no ORF3a mutation is found in the BA.1 variant or a single T223I ORF3a mutant is found in BA.2 and BA.3 variants (Tzou et al., 2020). Most interestingly, all three Omicron variants were first detected at approximately the same time and from the same place (Desingu et al., 2022). Thus, they should have equal chances to spread. However, only BA.1 variant is spreading very rapidly and it is now the most predominant viral variant in the world, and it also shows significantly attenuated viral virulence with reduced death rate and hospitalization (Abdullah et al., 2021; Espenhain et al., 2021). Although BA.2 and BA.3 variants are also attenuated, but they somehow spread much slower than BA.1. There is a total of seven mutations shared by BA.2 and BA.3 that are distinct from BA.1. One of the mutations is T223I of ORF3a that resides at the junction of β7 and β8 sheets. It is unknow at present whether the lack of ORF3a mutation in BA.1 or the T223I mutation in BA.2 and BA.3 have any functional relevance to viral transmission and disease severity of COVID-19, it would be interesting to investigate whether there is an association of viral attenuation (Abdullah et al., 2021; Desingu et al., 2022) and viral transmission observed in the Omicron variants with the lack of ORF3a mutation or the emergence of the T223I mutation.

## Final Remarks and Future Studies

We have learned a great deal about the function of SARS-CoV-2 ORF3a and its importance in viral pathogenesis and COVID-19 since the beginning of this pandemic. We have also confirmed some of ORF3a functionalities by comparing the studies of SARS-CoV and SARS-CoV-2 due to their sequence and structural similarities (Figure 1). While SARS-CoV-2 ORF3a is functionally similar to SARS-CoV ORF3a in many ways, SARS-CoV-2 ORF3a has clearly evolved and adapted subtle but new activities.

SARS-CoV-2 is a viroporin that forms calcium ion channels or interferes with ion channel activities of host plasma and endomembranes. Although ORF3a is an accessory protein, it plays important roles in virus reproduction in conjunction with E protein, and it exerts its effect throughout the viral life cycle (Figure 2), including viral uptake through endocytic pathways, DMV-associated viral transcription and replication on RTC, and viral release through exocytosis. Its expression in infected cells triggers cellular innate and pro-inflammatory immune responses that can induce a cytokine storm, especially under hypoxic conditions, by activating NLRP3 inflammasomes, HMGB1 and HIF-1α to promote the pro-inflammatory cytokine and chemokine production through NF-κB, TLR3, or TLR4-mediated pathways (Figure 4). Consequently, it leads to various forms of cell death including apoptosis, necrosis, and pyroptosis that contribute to tissue damage, COVID-19 and post-COVID morbidity.

Many of the SARS-CoV-2 ORF3a functions need further investigation. It remains unclear whether SARS-CoV-2 ORF3a’s ion channel activity is an intrinsic activity or if ORF3a interferes with an endogenous ion channel. While ORF3a continues to mutate, resulting in many emerging new mutant variants, there is no clear indication how the new ORF3a mutations affect its functions or their impact on viral pathogenesis and severity of COVID-19. It would be interesting to test whether SARS-CoV-2 ORF3a is directly involved in the activation of NLRP3 inflammasomes and participates in the induction of a cytokine storm especially under hypoxic conditions. Since cellular oxidative stress and inflammation cause tissue damage and various post-COVID conditions such as pulmonary fibrosis and “brain fog,” it would be important to study exactly how ORF3a exerts its effects on those conditions so that we could design ways to alleviate associated symptoms. Because SARS-CoV-2 ORF3a is a SARS-specific protein and its functions are associated with viral pathogenesis, viral reproduction, and COVID-19, ORF3a may be an appropriate therapeutic target to reduce viral production, alleviate symptoms of COVID-19 and post-COVID conditions. In addition, ORF3a-induced cell death is a highly conserved cellular response among fission yeast (Zhang et al., 2022), *Drosophila* (Yang et al., 2020), and humans (Ren et al., 2020), suggesting that it impacts fundamental cell death pathways. Therefore, ORF3a-induced cell death could provide a measurable endpoint to target ORF3a-induced adverse and cytopathic effects and to facilitate high-throughput screening of antiviral drugs against ORF3a (Zhao, 2017; Zhang et al., 2022). Although various inhibitors are described to inhibit ORF3a-mediated activities, no specific SARS-CoV-2 ORF3a inhibitor has been reported to target ORF3a directly or to inhibit all its activities, a prerequisite of a *bona fide* ORF3a inhibitor.

## Author Contributions

RZ wrote the manuscript. JZ, RZ, and AE generated the figures. JS, QT, MN, VG, AE, and JZ provided critiques and assisted in the writing and revision of the manuscript. All authors contributed to the article and approved the submitted version.

## Funding

This study was supported in part by grants from NIH R21 AI129369, NIH R01 GM127212/AI150459, VA BLR&D I01BX004652 and an intramural funding from the University of Maryland Medical Center (RZ). JS is supported by grants from the Department of Veterans Affairs (RR&D I01RX003060; BLR&D 1I01BX004652), the Department of Defense (SC170199), the National Heart, Lung and Blood Institute (R01HL082517), and the NINDS (R01NS102589; R01NS105633). VG is supported by NIH R01NS107262. QT is supported by NIH/NIAID and G12MD007597. The contents do not represent the views of the U.S. Department of Veterans Affairs or the United States Government.

## Conflict of Interest

The authors declare that the research was conducted in the absence of any commercial or financial relationships that could be construed as a potential conflict of interest.

## Publisher’s Note

All claims expressed in this article are solely those of the authors and do not necessarily represent those of their affiliated organizations, or those of the publisher, the editors and the reviewers. Any product that may be evaluated in this article, or claim that may be made by its manufacturer, is not guaranteed or endorsed by the publisher.
